# Editorial: Innate Anti-Tumor Immune Responses in Solid and Hematological Malignancies: From Basic Research to Clinical Applications

**DOI:** 10.3389/fimmu.2022.957119

**Published:** 2022-06-16

**Authors:** Anne-Sophie Chretien, Nicolas Dulphy, Emanuela Marcenaro

**Affiliations:** ^1^ Team Immunity and Cancer, Centre de Recherche en Cancérologie de Marseille (CRCM), Inserm U1068, CNRS UMR7258, Institut Paoli-Calmettes, Aix-Marseille University UM105, Marseille, France; ^2^ Immunomonitoring Department, Institut Paoli-Calmettes, Marseille, France; ^3^ Université Paris Cité, Institut de Recherche Saint Louis, Inserm U1160, Paris, France; ^4^ Laboratoire d'Immunologie et d'Histocompatibilité, Assistance Publique-Hôpitaux de Paris, Hôpital Saint-Louis, Paris, France; ^5^ Department of Experimental Medicine, University of Genova, Genova, Italy

**Keywords:** innate immunity, natural killer cells, hematological malignancies, solid tumor, viral infection, immunotherapy

The last decade has been marked by an accumulation of compelling evidence highlighting the importance of restoring functional immunity to stabilize or reverse cancer disease. Therapeutic strategies based on the manipulation of immunity allow obtaining complete remission rates never equaled with conventional chemotherapies and targeted therapies. In addition to the obvious role of adaptive immunity in tumor elimination processes, innate immune cells also contribute to tumor control and/or regression. Among the archetypal examples, we can mention NK cells, γδT cells, NKT cells as well as some populations of non-NK ILCs.

Innate immunity, in particular NK cells, plays a central role in the early stages of tumorigenesis, either directly by eliminating transformed cells, or indirectly by eliminating pathogens that promote tumor transformation. Reminiscent of the critical role of NK cells in managing viral infections as well as tumor progression, their control of EBV infections reduces the risk to develop EBV-induced malignancies in KIR haplotype B carriers. The work of Jiang et al. provides further arguments in favor of the central role of NK cells in this situation, with an imbalance of KIR haplotype B carriers among patients with EBV+ Hodgkin lymphoma. More recently, several data provided evidence that innate cells possess anti-SARS-CoV-2 activities, that can be compromised in patients with COVID-19. In this context, a dysfunctional state of tumor-associated NK cells has been described, including an increase in the expression of NKG2A, TIGIT and PD-1 immune checkpoints (ICs), and the application of the IC inhibitors (ICI) to rescue NK cells' activity thus facilitating viral control has been suggested. Notably, the immunomodulatory effects of ICI can modify the patients’ immune system function in SARS-CoV-2 infection. ICI can blockade the inhibitory signaling of ICs in both T and NK cells and augments the immune system response in COVID-19 patients. However, ICI may also increase the risk of cytokine release syndrome. In this context, the work by Rutigliani et al. provides evidence that the acute autoinflammatory response observed during SARS-CoV-2 infection could be due to the disruption of the PD-1/PD-L1 interaction and can lead to a poor prognosis.

Besides the role of innate immunity on tumor prevention, innate response upon treatment with chemotherapy, targeted therapy, radiotherapy or cell therapy, are cornerstones in supporting long-term complete remissions. Several recent examples linked survival rates with NK cell or γδ T cells parameters (Barros et al.). In line, Hussein et al. studied the impact of activating receptor gene variants on NK cell receptor expression and survival in cohort of AML patients. The authors found that some single nucleotide polymorphisms of genes encoding the NK-cell triggering receptors NKG2D and DNAM-1 were associated with favorable clinical outcomes in AML patients treated with IL-2 and histamine.

Interestingly, innate immune alterations are often uncorrelated with T-cell defects and may therefore be integrated into immune signatures based on variables related to conventional T cell functions. T cell alterations have been associated with prognosis or response to therapy, with an example of application detailed in the article by Qian et al. In this work, the team developed a 15-gene immune signature, including antigen presenting molecule machinery and chemokine receptors, associated with response to neoadjuvant chemoradiotherapy in colorectal cancer. Non-responders displayed increased transcriptomic signatures of naïve CD4, exhausted T cells and Th1, and decreased transcriptomic signature of Tfh. Some T-cell parameters being poorly correlated with innate immune parameters, combinatorial approaches are expected to improve the quality of prediction of immune signatures based on T cell transcriptomic signatures or phenotypic profiles.

ICI constituted a revolution in the strategies for cancer treatment. Guolo et al. suggest a significant role of mature KIR+ NK cells in patients with refractory Hodgkin lymphoma treated with nivolumab and autologous lymphocytes infusions, besides the good tolerability and promising complete remission rates. This finding is in line with previous work showing a beneficial effect of ICIs on NK cell-mediated anti-tumor immunity in Hodgkin lymphoma. This work also reinforces the conclusions of previous work highlighting the importance of NK cell maturation homeostasis in hematological malignancies.

ICs are also expressed by ILCs. In the work of Cristiani et al., nivolumab impacted the relative proportions of ILC subsets, with a contraction of total peripheral ILCs, as well as an expansion of CD117- ILC2s, a subset of mature ILC2 that secretes type 2 cytokines. Interestingly, a low frequency of CD117- ILC2 after treatment with nivolumab was associated with improved survival.

Another recent work has identified candidate targets for next-generation immunotherapy that may eventually complement the existing therapeutic arsenal, including molecules expressed by innate immune cells. Among these, the LLT1 CD161 axis is particularly interesting given its association with prognosis in most cancers (Braud et al.). As with other immune targets under development, CD161 is expressed by both innate and adaptive populations, including NK cells, γδ T cells and ILCs, and it can be hypothesized that innate immunity will directly or indirectly participate to therapeutic response.

Taken together, the articles gathered in the present topic provide a panel of examples of recent advances of basic and translational research on lymphoid innate anti-tumor immune responses, which are progressively bridging the gap between fundamental knowledge and potential clinical applications. Harnessing innate immune cells against tumor cells is probably the next challenge that will hold its promises in clinical settings. The current pre-clinical and clinical development of antibodies likely to restore innate immunity as well as the extensive development of CAR-NK cells and CAR-γδ T cells already provided encouraging results, with good tolerance and signs of clinical activity, and are expected to find applications in the next coming years(Barros et al.). Finally, alterations of innate immune populations are an important source of biomarkers to discriminate the patients most likely to respond to chemotherapies and immunotherapies and, moreover, it also informs us about the mechanisms of primary and secondary resistance to these drugs, whose target populations were initially the conventional T cells.

## Author Contributions

All authors listed have made a substantial, direct, and intellectual contribution to the work and approved it for publication.

## Funding

The research leading to these results has received funding from AIRC under IG 2021 – ID. 26037 project – P.I. Marcenaro Emanuela. Additional grants from. University of Genova: Compagnia di San Paolo (2019.866) (to EM).

## Conflict of Interest

The authors declare that the research was conducted in the absence of any commercial or financial relationships that could be construed as a potential conflict of interest.

## Publisher’s Note

All claims expressed in this article are solely those of the authors and do not necessarily represent those of their affiliated organizations, or those of the publisher, the editors and the reviewers. Any product that may be evaluated in this article, or claim that may be made by its manufacturer, is not guaranteed or endorsed by the publisher.

